# Effects of Resveratrol, Quercetin and Esculin on Inflammatory Injury and Apoptosis of IPEC-J2 Cells Induced by Soybean 7S Globulin

**DOI:** 10.3390/vetsci13101048

**Published:** 2026-10-07

**Authors:** Ruoxu Wang, Yiming Xie, Renwu Zhou, Jie Zhang, Hongrui Guo, Ya Wang, Junliang Deng, Huidan Deng

**Affiliations:** 1 College of Veterinary Medicine, Sichuan Agricultural University, Chengdu 611130, China; 2 Sichuan Animal Science Academy, Chengdu 610066, China; 3 Key Laboratory of Animal Diseases and Environmental Hazards of Sichuan Province, Sichuan Agricultural University, Chengdu 611130, China

**Keywords:** soybean 7S globulin, porcine intestinal epithelial cells, bioactive phytochemicals, inflammatory injury, apoptosis

## Abstract

Soybean is the primary protein source for weaned piglets; however, soybean 7S globulin can induce allergic enteritis and diarrhea and may cause death. Antibiotic feed additives have been used to control these symptoms, but their use increases the risk of antimicrobial resistance, highlighting an urgent need for alternatives. Polyphenol-rich phytochemicals such as resveratrol, quercetin, and esculin exhibit antioxidant, anti-inflammatory, and immunomodulatory activities; however, whether they protect against soybean 7S globulin-induced intestinal epithelial injury, and through which mechanisms, remains unclear. This study investigated how soybean 7S globulin injures porcine jejunal epithelial (IPEC-J2) cells and evaluated the protective effects of these three compounds against inflammatory injury and apoptosis. Cell viability was assessed using the CCK-8 assay; inflammatory cytokines and oxidative stress indicators were measured by ELISA and microplate assays; apoptosis was determined by flow cytometry; and gene and protein expression was analyzed by qRT-PCR and Western blotting. Soybean 7S globulin (5 mg/mL) reduced IPEC-J2 cell viability, impaired antioxidant defenses, and induced inflammatory injury and apoptosis via the NF-κB/MAPK pathway. All three compounds alleviated these effects; notably, 0.625 mg/L resveratrol, 2.5 mg/L quercetin, and 20 mg/L esculin exerted the strongest protection by attenuating oxidative stress, regulating apoptosis-related gene expression, and mitigating apoptosis.

## Simple Summary

Soybean is the main protein source in feed for newly weaned piglets; however, soybean 7S globulin, a major soybean allergen, can damage intestinal epithelial cells, trigger inflammatory diarrhea, and impair piglet health. This issue is closely linked to livestock production safety and public health from a One Health perspective, and conventional antibiotic treatments carry the risks of drug residues and antimicrobial-resistant bacteria, which threaten both animal and human health. This study investigated whether three natural plant-derived compounds—resveratrol, quercetin, and esculin—could protect piglet intestinal epithelial cells from such damage. We found that soybean 7S globulin reduced cell viability, induced oxidative injury and inflammation, and promoted apoptosis. All three compounds alleviated these detrimental effects, with specific doses providing the strongest protection by modulating key signaling pathways involved in inflammation and apoptosis. These findings support the development of natural, residue-free feed additives to safeguard piglet intestinal health, reduce antibiotic use, and promote healthy and sustainable livestock production.

## 1. Introduction

Soybean protein has high biological value with a balanced essential amino acid profile and is widely used as a cost-effective, plant-derived protein source to replace animal-based protein in piglet diets, supporting muscle growth and intestinal development in weaned piglets. During weaning, piglets transition from highly digestible liquid milk to a solid diet containing complex proteins [1]. This drastic change in dietary composition poses a considerable challenge to the fragile intestinal tract of weaned piglets, which require highly digestible, high-quality proteins to alleviate weaning stress and meet their nutritional needs.

However, soybean protein, the main protein source in feed for weaned piglets, contains various anti-nutritional factors that not only impair nutrient absorption but also cause diarrhea and even death in piglets [2]. Among these factors, soybean 7S globulin (β-conglycinin), a trimeric glycoprotein with a molecular weight of 140–180 kDa, is the major soybean allergen and is the most likely to induce intestinal allergic reactions, resulting in diarrhea and death in piglets [3]. Its pathogenic mechanisms are mainly associated with IgE-mediated type I hypersensitivity [4], immune complex-mediated type III hypersensitivity [5], T lymphocyte-mediated type IV hypersensitivity [6], oxidative stress, inflammatory responses, and apoptosis. On the one hand, soybean 7S globulin induces allergic reactions that lead to inflammatory injury. Peng et al. [7] found that 5 mg/mL soybean 7S globulin reduced the viability of IPEC-J2 cells and activated the NF-κB signaling pathway, thereby causing inflammatory damage. Our previous experiments also confirmed that 5 mg/mL soybean 7S globulin significantly upregulated pro-inflammatory cytokine levels and significantly reduced anti-inflammatory cytokine levels in the culture medium of jejunal epithelial cells from weaned piglets [8]. On the other hand, the production of pro-inflammatory cytokines induced by soybean 7S globulin is accompanied by the excessive generation of reactive oxygen species (ROS). Through the Fenton reaction, ROS cause lipid peroxidation of cellular membranes, leading to the accumulation of malondialdehyde (MDA), the end product of lipid peroxidation, which directly participates in the inflammatory process, exacerbates systemic inflammation, and causes oxidative damage [9]. Notably, the excessive ROS production induced by soybean 7S globulin is also a key factor underlying the inhibition of cell proliferation and the activation of apoptosis. ROS-induced apoptosis may be mediated by activation of NF-κB [10] and mitogen-activated protein kinase (MAPK) [11] signaling pathways and by mitochondrial damage [12].

The NF-κB/MAPK signaling pathway plays a crucial role in regulating genes associated with oxidative stress, inflammation, and apoptosis [13]. Peng et al. [14] reported that 5 mg/mL soybean 7S globulin significantly increased the levels of pro-inflammatory factors, upregulated apoptosis-related genes, and activated the NF-κB/MAPK signaling pathway, whereas inhibition of this pathway significantly alleviated the above changes, suggesting that soybean 7S globulin induces inflammatory responses and apoptosis through the NF-κB/MAPK signaling pathway. Sun et al. [15] found that 5 mg/mL soybean 7S globulin reduced cell viability and interleukin-10 (IL-10) content, increased the contents of nitric oxide (NO), tumor necrosis factor-α (TNF-α), and interferon-γ (IFN-γ), and upregulated the mRNA and protein expression levels of NF-κB, inducible nitric oxide synthase (iNOS), c-Jun N-terminal kinase (JNK), and p38 mitogen-activated protein kinase (p38 MAPK). Peng et al. [16] also found that 5 mg/mL soybean 7S globulin significantly increased the contents of NO and IL-10, significantly upregulated the expression of Caspase-3 and the Bax/Bcl-xl ratio, and promoted the phosphorylation of p38 and JNK proteins; these effects were significantly alleviated by the addition of NF-κB/MAPK signaling pathway inhibitors.

At present, antibiotics and other additives are commonly used to control diarrhea in piglets. However, the widespread use of antibiotics and other chemical drugs in production has led to varying degrees of drug residues and other drawbacks [17]. Consequently, increasing attention has been directed toward the development of traditional Chinese medicines, which exhibit minimal toxic side effects, leave no residues, and are unlikely to induce drug-resistant microorganisms [18]. Resveratrol (RES; 3,5,4′-trihydroxystilbene) is one of the most abundant polyphenols in red grapes, and multiple studies have demonstrated its strong potential for promoting growth performance, exerting antioxidant and anti-inflammatory effects, and regulating the intestinal barrier in animals [19]. Yi et al. [20] showed that dietary resveratrol supplementation significantly enhanced intestinal antioxidant enzyme activities, promoted the expression of intestinal barrier genes, and improved intestinal morphology and growth performance in weaned piglets. Quercetin, a representative flavonoid abundant in fruits and vegetables such as onions and apples, exists predominantly in glycosidic forms and exhibits multiple biological activities, including promoting the proliferation of porcine intestinal epithelial cells, modulating the growth of certain gut microbiota, and regulating the expression of related functional genes. Quercetin is metabolized and excreted mainly through the liver, intestine, and kidneys, with substantial accumulation in the colon [21]. Xing [22] reported that quercetin effectively alleviated intestinal stress syndrome and relieved symptoms such as diarrhea and mild gastrointestinal inflammation. Esculin has been reported to exert anti-oxidative and anti-inflammatory effects via the MAPK pathway, inhibiting pro-inflammatory factors and promoting anti-inflammatory factors [23]. Collectively, resveratrol, quercetin, and esculin possess anti-inflammatory, antioxidant, anti-apoptotic, and immunomodulatory properties and have shown beneficial effects in models of inflammatory bowel disease, suggesting that these bioactive phytochemicals may have therapeutic potential against soybean 7S globulin-induced allergic enteritis and diarrhea in weaned piglets.

The intestinal epithelial monolayer functions not only as a physical barrier but also as a critical defense that prevents macromolecules and pathogenic microorganisms in the intestinal lumen from invading the underlying mucosa. IPEC-J2 cells, isolated from the jejunum of neonatal unsuckled piglets, are a non-transformed, non-tumorigenic epithelial cell line of small-intestinal origin [24]. It therefore serves as an ideal in vitro model for studying epithelial transport, interactions with intestinal bacteria and probiotics, and the effects of nutrients and other feed components on permeability, metabolic activity, and other aspects of epithelial function.

Despite the reported benefits of resveratrol, quercetin, and esculin, whether these compounds can protect porcine intestinal epithelial cells against soybean 7S globulin-induced injury—and through which mechanisms—has not been systematically investigated, representing a clear research gap. Therefore, this study was conducted to investigate the effects of resveratrol, quercetin, and esculin on cell viability, inflammatory responses, oxidative stress, and apoptosis in IPEC-J2 cells injured by soybean 7S globulin, and to explore the underlying protective mechanisms. Our findings provide a theoretical basis for the potential application of resveratrol, quercetin, and esculin in the prevention and treatment of allergic enteritis in weaned piglets caused by soybean 7S globulin.

## 2. Materials and Methods

### 2.1. Reagents and Drugs

Soybean 7S globulin (purity 83 ± 3%; batch No. 20211118) was purchased from Shanghai Yingwan Biotechnology Co., Ltd. (Shanghai, China). Before use, the commercially obtained lyophilized powder was dissolved directly in the cell culture medium to a final concentration of 5 mg/mL and sterilized by filtration through a 0.22 μm membrane. Resveratrol (purity ≥ 98%; product No. B20570), quercetin (purity ≥ 98%; product No. B20527), and esculin (purity ≥ 98%; product No. B20989) were purchased from Shanghai Yuanye Biotechnology Co., Ltd. (Shanghai, China).

### 2.2. Cell Culture and Treatments

The porcine jejunal epithelial cell line IPEC-J2 was purchased from Shanghai Yingwan Biotechnology Co., Ltd. (Shanghai, China). IPEC-J2 is a non-transformed, non-tumorigenic porcine jejunal epithelial cell line that retains the morphological and functional characteristics of intestinal epithelial cells and is therefore widely used to investigate intestinal epithelial injury induced by feed-derived allergens. Because this study used only a commercially available immortalized cell line and did not involve live animals or human subjects, ethical approval was not required.Cells were cultured in DMEM/F12 (HyClone; Logan, UT, USA) supplemented with 10% fetal bovine serum (FBS; TransGen Biotech, Beijing, China), penicillin–streptomycin (100×; Beijing Solarbio Science & Technology Co., Ltd., Beijing, China) at 37 °C in a humidified atmosphere containing 5% CO_2_.

Prior to the formal experiments, a dose-screening pre-assay was performed to identify the non-cytotoxic concentrations of resveratrol, quercetin, and esculin. Three concentrations of each compound that had no adverse effect on cell viability were selected for subsequent experiments. All compounds were dissolved in dimethyl sulfoxide (DMSO), and the final DMSO concentration in all culture groups was maintained below 0.1% (*v*/*v*) to exclude solvent-induced cytotoxicity; an equivalent concentration of DMSO was added to the blank control and model groups as the vehicle control.

For the formal experiments, a total of 14 groups were established: a blank control group; a model group treated with 5 mg/mL soybean 7S globulin; nine treatment groups co-incubated with 5 mg/mL soybean 7S globulin and graded concentrations of each compound (0.156, 0.313, and 0.625 mg/L resveratrol; 0.625, 1.25, and 2.5 mg/L quercetin; and 10, 15, and 20 mg/L esculin); and three compound-only control groups treated with the highest selected concentration of each compound in the absence of soybean 7S globulin. In all treatment groups, the compounds were applied together with soybean 7S globulin (co-incubation) for 24 h; thus, the present study evaluated the protective rather than the therapeutic effects of these compounds. Each experiment was independently repeated at least three times (*n* = 3 independent experiments), with three technical replicates per group.

### 2.3. CCK-8 Assay

IPEC-J2 cells were seeded into 96-well plates and treated with soybean 7S globulin, resveratrol, quercetin, or esculin for 24 h as described above. CCK-8 reagent (Sangon Biotech (Shanghai) Co., Ltd., Shanghai, China) 10 μL per well was then added, and the cells were incubated at 37 °C with 5% CO_2_ for 4 h. The absorbance was measured at 450 nm using a microplate reader (Thermo Fisher Scientific, Waltham, MA, USA), and cell viability was calculated according to previously described protocols [25]. Cell viability (%) = [(OD of the experimental group − OD of the blank group)/(OD of the control group − OD of the blank group)] × 100%.

### 2.4. ELISA Assay

Cells were seeded into 96-well plates and subjected to the designated treatments for 24 h. Cell culture supernatants were then collected, and the levels of iNOS, the anti-inflammatory cytokines IL-4 and IL-10, and the pro-inflammatory cytokines IL-6, IL-1β, and TNF-α were measured using commercial ELISA kits (Jiangsu Enzyme Immunoassay Industry Co., Ltd., Jiangsu, China) according to the manufacturer’s instructions. Absorbance was read at 450 nm using a microplate reader [26].

### 2.5. Measurement of Cellular Oxidative Indicators and Cytokine Contents by Microplate Assay

At the end of the treatments, cell pellets were collected, and the concentrations of MDA, GSH, and NO and the activity of superoxide dismutase (SOD) were measured using commercial microassay kits (Suzhou Keming Biotechnology Co., Ltd., Suzhou, China) according to the manufacturer’s instructions.

### 2.6. Detection of Apoptosis Rate and ROS Levels by Flow Cytometry

IPEC-J2 cells were seeded into 25 cm^2^ cell culture flasks at a density of 1 × 10^5^ cells/mL. After co-treatment with the drug and soybean 7S globulin concentrations described in Section 2.2 for 24 h, cell pellets were collected and stored on ice. For ROS level detection: 100 μL of cell pellet was transferred to a flow cytometry tube, added with 1 mL of 25 μM DCFH-DA probe (Sigma-Aldrich, St. Louis, MO, USA), and incubated at 37 °C for 30 min. After centrifugation at 300 g for 5 min, the supernatant was discarded. The cells were resuspended in 1 mL of PBS, centrifuged at 300 g for 5 min again, and the supernatant was removed. Subsequently, 400 μL of PBS was added to resuspend the cells, and the ROS level was immediately detected using a flow cytometer (Beckman Coulter, Brea, CA, USA). For apoptosis rate detection, 100 μL of cell suspension was resuspended in 200 μL of freshly prepared 1× binding buffer, then 5 μL of Annexin V-FITC Apoptosis Detection Kit (eBioscience, San Diego, CA, USA) fluorescent dye was added and incubated at room temperature in the dark for 10 min. Then 10 μL of PI was added for 5 min of staining (room temperature, dark condition). After adding 400 μL of PBS to resuspend the cells, the apoptosis rate was immediately detected by flow cytometry.

### 2.7. Quantitative Real-Time PCR (qRT-PCR)

Total RNA was extracted using a total RNA extraction kit (Accurate Biotechnology (Hunan) Co., Ltd., Changsha, China) and reverse-transcribed into complementary DNA (cDNA) using a reverse transcription kit (FOREGENE, Chengdu, China) according to the manufacturer’s instructions. The synthesized cDNA was diluted five-fold and used as the template for subsequent qRT-PCR.

qRT-PCR was performed on a CFX96 Real-Time PCR System (Bio-Rad, Hercules, CA, USA) using a SYBR Green qPCR kit according to the manufacturer’s protocol. The total reaction volume was 20 μL, consisting of 10 μL of 2× SYBR Green Pro Taq HS Premix qPCR Kit (Accurate Biotechnology (Hunan) Co., Ltd., Changsha, China) master mix, 2 μL of diluted cDNA template, 0.5 μL of 40× Dilution Buffer, 0.4 μL of forward primer (10 μM), 0.4 μL of reverse primer (10 μM), and 6.7 μL of RNase-free water. The amplification conditions were as follows: initial denaturation at 95 °C for 30 s, followed by 40 cycles of denaturation at 95 °C for 5 s and annealing/extension at 60 °C for 30 s. Melting curve analysis was performed after amplification to verify the specificity of the PCR products. Each sample was analyzed in three technical replicates, and three independent biological replicates were performed.

The relative mRNA expression levels of the target genes were calculated using the 2^−ΔΔCt^ method. Prior to the formal experiments, all primer pairs were validated by standard curve analysis, and the amplification efficiencies ranged from 90% to 110%. β-actin was used as the reference gene; the limitations associated with the use of a single reference gene were taken into account when interpreting the transcriptional results. Primers were synthesized by Sangon Biotech (Shanghai) Co., Ltd. (Shanghai, China), and their sequences are listed in Table 1.

### 2.8. Western Blot Assay

After the designated treatments, the cells were washed two to three times with ice-cold PBS, and residual liquid was thoroughly aspirated. The cells were scraped from the culture plates with a cell scraper and lysed in RIPA lysis buffer (Beijing Solarbio Science & Technology Co., Ltd., Beijing, China) supplemented with protease and phosphatase inhibitor cocktails (Servicebio, Wuhan, China) immediately before use. Lysis was performed on ice for 30 min with periodic pipetting to ensure complete cell lysis. The lysates were centrifuged at 12,000 r/min for 10 min at 4 °C, and the supernatants were collected as total protein samples.

Protein concentrations were determined using a BCA (Thermo Fisher Scientific, Waltham, MA, USA) protein assay kit (Thermo Fisher Scientific). The protein samples were mixed with 5× reducing loading buffer at a volume ratio of 4:1, denatured by boiling in a water bath for 15 min, and stored at −20 °C until analysis.

Equal amounts of protein (30 μg per lane) were separated by sodium dodecyl sulfate-polyacrylamide gel electrophoresis (SDS-PAGE) using a Mini-PROTEAN TGX system (Bio-Rad, Hercules, CA, USA) with a 5% stacking gel and a 10% separating gel. Electrophoresis was run at a constant voltage of 90 V for the stacking gel and 150 V for the separating gel and was terminated when the bromophenol blue dye front reached approximately 1 cm from the bottom of the gel.

Following electrophoresis, the proteins were electrotransferred onto 0.45 μm PVDF membranes (Merck Millipore, Billerica, MA, USA), which had been activated with methanol for 2 min. Electrotransfer was conducted at a constant current of 300 mA for 30 min in an ice bath to prevent overheating. After transfer, the membranes were briefly rinsed with Tris-buffered saline containing 0.1% Tween 20 (TBST) and then blocked with 5% (*w*/*v*) non-fat dry milk (Solarbio) (for total protein detection) or 5% (*w*/*v*) bovine serum albumin (BSA; for phosphorylated protein detection) on an orbital shaker for 30 min at room temperature. The membranes were then incubated with appropriately diluted primary antibodies at 4 °C overnight with gentle shaking. The primary antibodies used were as follows: rabbit anti-NF-κB p65 (GB11997; 1:1000), rabbit anti-phospho-NF-κB p65 (Ser536) (1:1000), rabbit anti-p38 MAPK (GB114685-100; 1:1000), rabbit anti-phospho-p38 MAPK (Thr180/Tyr182) (GB153380; 1:1000), rabbit anti-JNK (GB114321-100; 1:1000), rabbit anti-phospho-JNK (Thr183/Tyr185) (GB15018; 1:500), and rabbit anti-GAPDH (GB15004; 1:5000) (all from Servicebio, Wuhan, China).

After primary antibody incubation, the membrane was rinsed rapidly three times with TBST and then washed on a decolorizing shaker, 5 min per wash, for a total of three washes. The HRP-conjugated goat anti-rabbit IgG (H + L) secondary antibody (GB23303; Servicebio, Wuhan, China) was diluted with TBST at 1:5000, added to the incubation chamber, and incubated on a shaker with gentle agitation at room temperature for 30 min. The membrane was rinsed rapidly three times with TBST and then washed on a decolorizing shaker, 5 min per wash, for a total of three washes. For chemiluminescent detection, ECL chemiluminescence kit (Wuhan Servicebio Technology Co., Ltd., Wuhan, China) reagents A and B were mixed at a 1:1 ratio immediately before use. The washed membranes were transferred to the imaging platform of a MyECL chemiluminescence imager (Thermo Fisher Scientific), fully covered with the ECL working solution, and imaged after 1 min of reaction according to the preset procedure.

Band intensities were quantified using ImageJ software (version 1.54g, National Institutes of Health, Bethesda, MD, USA). The relative expression of total target proteins was normalized to the internal reference GAPDH, and the relative expression of phosphorylated proteins was normalized to the corresponding total protein levels.

### 2.9. Data Analysis

All data are presented as the mean ± standard deviation (SD) of at least three independent experiments. Differences among groups were analyzed by one-way analysis of variance (ANOVA) followed by Tukey’s post hoc multiple-comparisons test using GraphPad Prism 9.0 (GraphPad Software, Inc., San Diego, CA, USA). A *p* value < 0.05 was considered statistically significant.

## 3. Results

### 3.1. Effects of Resveratrol, Quercetin, and Esculin on the Viability of IPEC-J2 Cells

**Figure 1:**
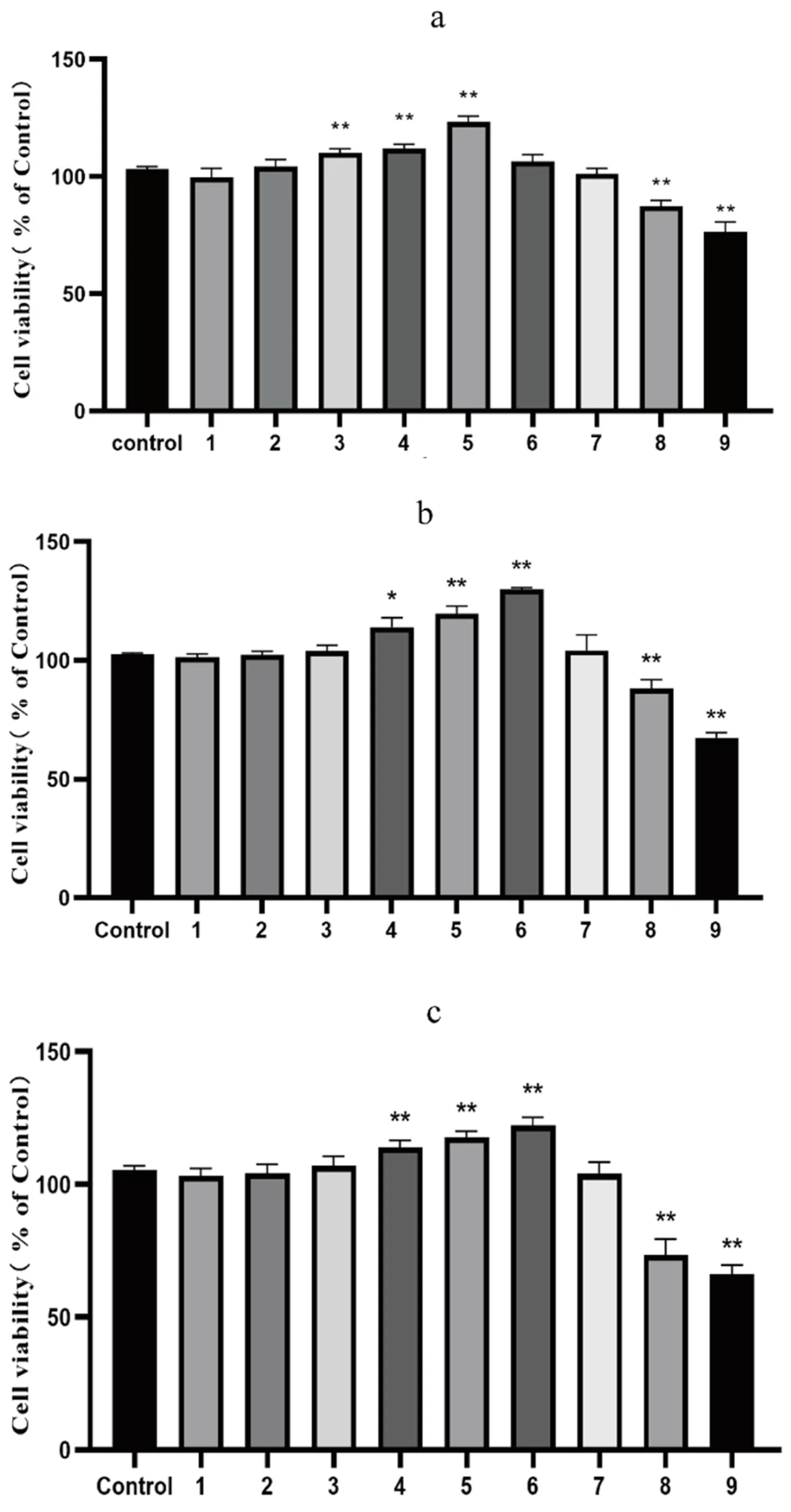
Effects of resveratrol, quercetin, and esculin on the viability of IPEC-J2 cells. Note: (**a**) resveratrol treatment groups; control: blank control group; lanes 1–9: 0.039, 0.078, 0.156, 0.313, 0.625, 1.25, 2.5, 5, and 10 mg/L, respectively. (**b**) Quercetin treatment groups; control: blank control group; lanes 1–9: 0.078, 0.156, 0.313, 0.625, 1.25, 2.5, 5, 10, and 20 mg/L, respectively. (**c**) Esculin treatment groups; control: blank control group; lanes 1–9: 1.25, 2.5, 5, 10, 15, 20, 30, 40, and 50 mg/L, respectively. * *p* < 0.05 and ** *p* < 0.01 vs. the control group.

The effects of resveratrol, quercetin, and esculin on the viability of IPEC-J2 cells are shown in Figure 1. All three compounds exhibited a biphasic effect on cell viability: within the safe concentration range, cell viability increased progressively with increasing concentration until reaching a peak, whereas beyond the critical concentration, cell viability declined as the concentration continued to rise. For resveratrol (Figure 1a), compared with the blank control group, resveratrol at 0.156, 0.313, and 0.625 mg/L significantly enhanced IPEC-J2 cell viability (*p* < 0.01), with the 0.625 mg/L group showing the strongest stimulatory effect; at 5 and 10 mg/L, cell viability was significantly lower than that of the control group (*p* < 0.01). For quercetin (Figure 1b), quercetin at 0.625 mg/L significantly increased cell viability compared with the blank control group (*p* < 0.05), and 1.25 and 2.5 mg/L produced more pronounced stimulatory effects (*p* < 0.01); at 10 and 20 mg/L, cell viability was significantly reduced (*p* < 0.01). For esculin (Figure 1c), esculin at 10, 15, and 20 mg/L significantly enhanced cell viability compared with the blank control group (*p* < 0.01), whereas at 40 and 50 mg/L, cell viability was significantly decreased (*p* < 0.01). Based on these findings, the formal experiments used resveratrol at 0.156, 0.313, and 0.625 mg/L (designated R1, R2, and R3), quercetin at 0.625, 1.25, and 2.5 mg/L (designated Q1, Q2, and Q3), and esculin at 10, 15, and 20 mg/L (designated E1, E2, and E3) for intervention in the soybean 7S globulin injury model.

### 3.2. Effects of Resveratrol, Quercetin, and Esculin on the Viability of IPEC-J2 Cells Treated with Soybean 7S Globulin

**Figure 2:**
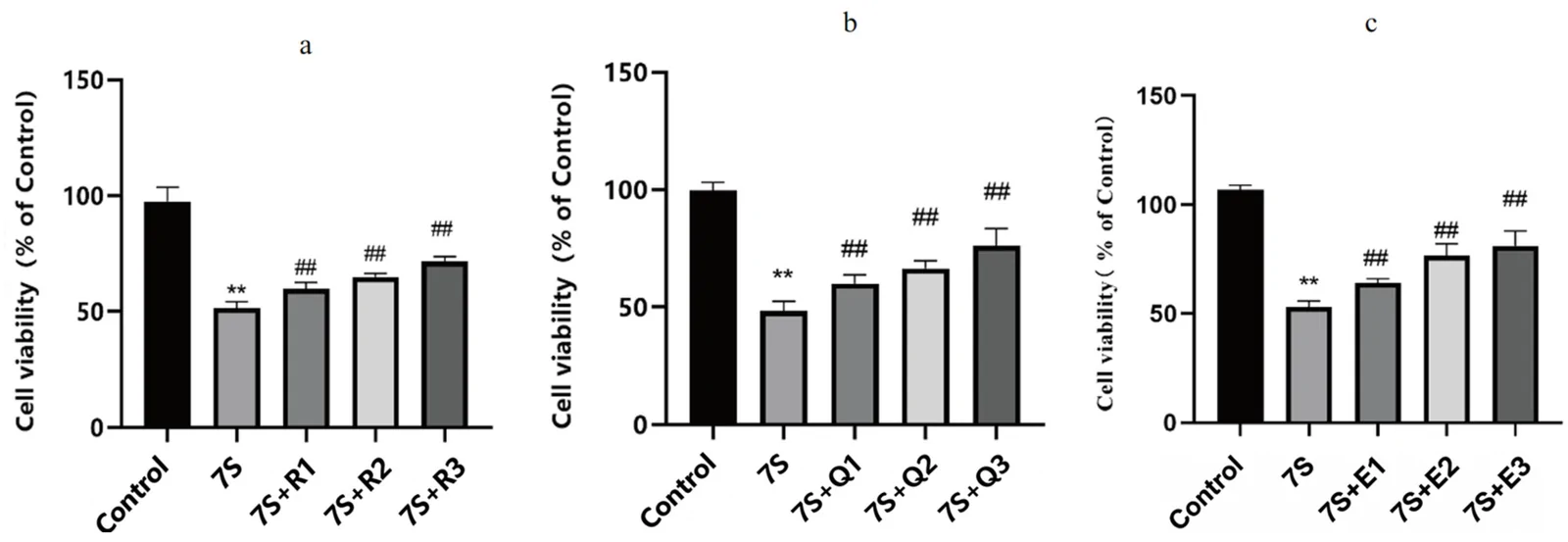
Effects of resveratrol, quercetin, and esculin on the viability of IPEC-J2 cells treated with soybean 7S globulin. Note: (**a**) Resveratrol treatment groups; (**b**) quercetin treatment groups; (**c**) esculin treatment groups. Control: cells without any compound or soybean 7S globulin; 7S: 5 mg/mL soybean 7S globulin alone; 7S + R1, 7S + R2, and 7S + R3: 5 mg/mL soybean 7S globulin plus 0.156, 0.313, and 0.625 mg/L resveratrol, respectively; 7S + Q1, 7S + Q2, and 7S + Q3: 5 mg/mL soybean 7S globulin plus 0.625, 1.25, and 2.5 mg/L quercetin, respectively; 7S + E1, 7S + E2, and 7S + E3: 5 mg/mL soybean 7S globulin plus 10, 15, and 20 mg/L esculin, respectively. ** *p* < 0.01 vs. the control group; ## *p* < 0.01 vs. the soybean 7S globulin model group.

Based on the dose-screening results described in Section 3.1, three non-cytotoxic concentrations of each compound were selected for the intervention experiments, and the results are shown in Figure 2. Compared with the blank control group, treatment with 5 mg/mL soybean 7S globulin for 24 h significantly reduced IPEC-J2 cell viability (*p* < 0.01). Compared with the soybean 7S globulin model group, all tested concentrations of resveratrol, quercetin, and esculin significantly increased cell viability (*p* < 0.05 or *p* < 0.01). Among the tested concentrations, 0.625 mg/L resveratrol, 2.5 mg/L quercetin, and 20 mg/L esculin yielded the highest numerical cell viability values, which were significantly greater than those of the corresponding low-concentration groups (*p* < 0.05); these three concentrations were therefore selected for the subsequent mechanistic experiments.

### 3.3. Effects of Resveratrol, Quercetin, and Esculin on the Inflammatory Response Induced by Soybean 7S Globulin in IPEC-J2 Cells

**Figure 3:**
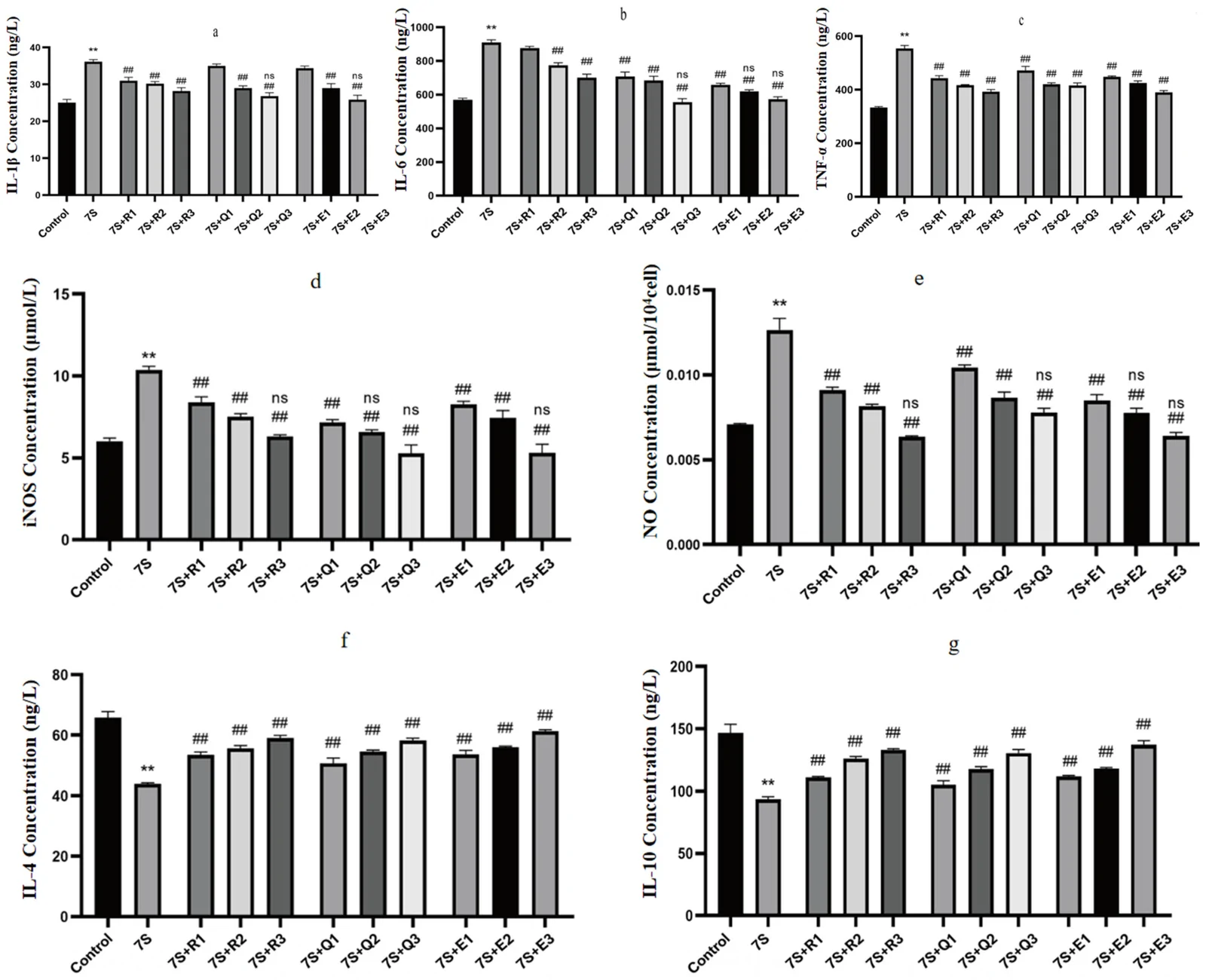
Effects of resveratrol, quercetin, and esculin on pro-inflammatory cytokines, intracellular iNOS and NO concentrations, and anti-inflammatory cytokines in IPEC-J2 cells treated with soybean 7S globulin. Note: (**a**) IL-1β concentration; (**b**) IL-6 concentration; (**c**) TNF-α concentration; (**d**) iNOS concentration; (**e**) NO concentration; (**f**) IL-4 concentration; (**g**) IL-10 concentration. ** *p* < 0.01 vs. the control group; ## *p* < 0.01 vs. the soybean 7S globulin model group; ns, not significant (*p* > 0.05).

The effects on pro-inflammatory cytokines are shown in Figure 3a–c. Compared with the control group, soybean 7S globulin challenge significantly elevated the levels of IL-1β, IL-6, and TNF-α (all *p* < 0.01). Compared with the soybean 7S globulin group, all three compounds significantly decreased the levels of these pro-inflammatory cytokines (*p* < 0.05 or *p* < 0.01).

As shown in Figure 3d,e, intracellular iNOS and NO concentrations were markedly increased by soybean 7S globulin (both *p* < 0.01) and were significantly reduced by resveratrol, quercetin, and esculin (all *p* < 0.01).

As shown in Figure 3f,g, soybean 7S globulin significantly suppressed the levels of the anti-inflammatory cytokines IL-4 and IL-10 (both *p* < 0.01), whereas all three compounds significantly restored the levels of these cytokines compared with the soybean 7S globulin group (*p* < 0.05 or *p* < 0.01).

### 3.4. Effects of Resveratrol, Quercetin, and Esculin on Soybean 7S Globulin-Induced Oxidative Stress in IPEC-J2 Cells

**Figure 4:**
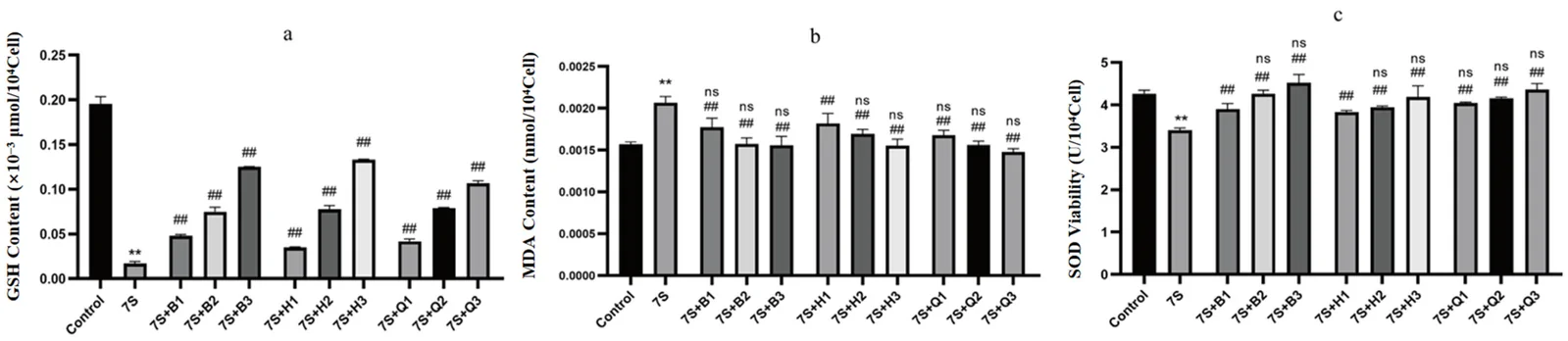
Effects of resveratrol, quercetin, and esculin on MDA and GSH concentrations and SOD activity in IPEC-J2 cells treated with soybean 7S globulin. Note: (**a**) GSH concentration; (**b**) MDA concentration; (**c**) SOD activity. ** *p* < 0.01 vs. the control group; ## *p* < 0.01 vs. the soybean 7S globulin model group; ns, not significant (*p* > 0.05).

The effects on oxidative stress indicators are shown in Figure 4. Compared with the control group, soybean 7S globulin challenge significantly increased the MDA concentration (*p* < 0.01), decreased the GSH concentration (*p* < 0.01), and reduced SOD activity (*p* < 0.01). Compared with the soybean 7S globulin group, all tested concentrations of resveratrol, quercetin, and esculin significantly decreased the MDA concentration (*p* < 0.05 or *p* < 0.01), increased the GSH concentration (*p* < 0.05 or *p* < 0.01), and enhanced SOD activity (*p* < 0.05 or *p* < 0.01).

### 3.5. Effects of Resveratrol, Quercetin, and Esculin on Intracellular ROS Generation and Apoptosis in IPEC-J2 Cells Induced by Soybean 7S Globulin

**Figure 5:**
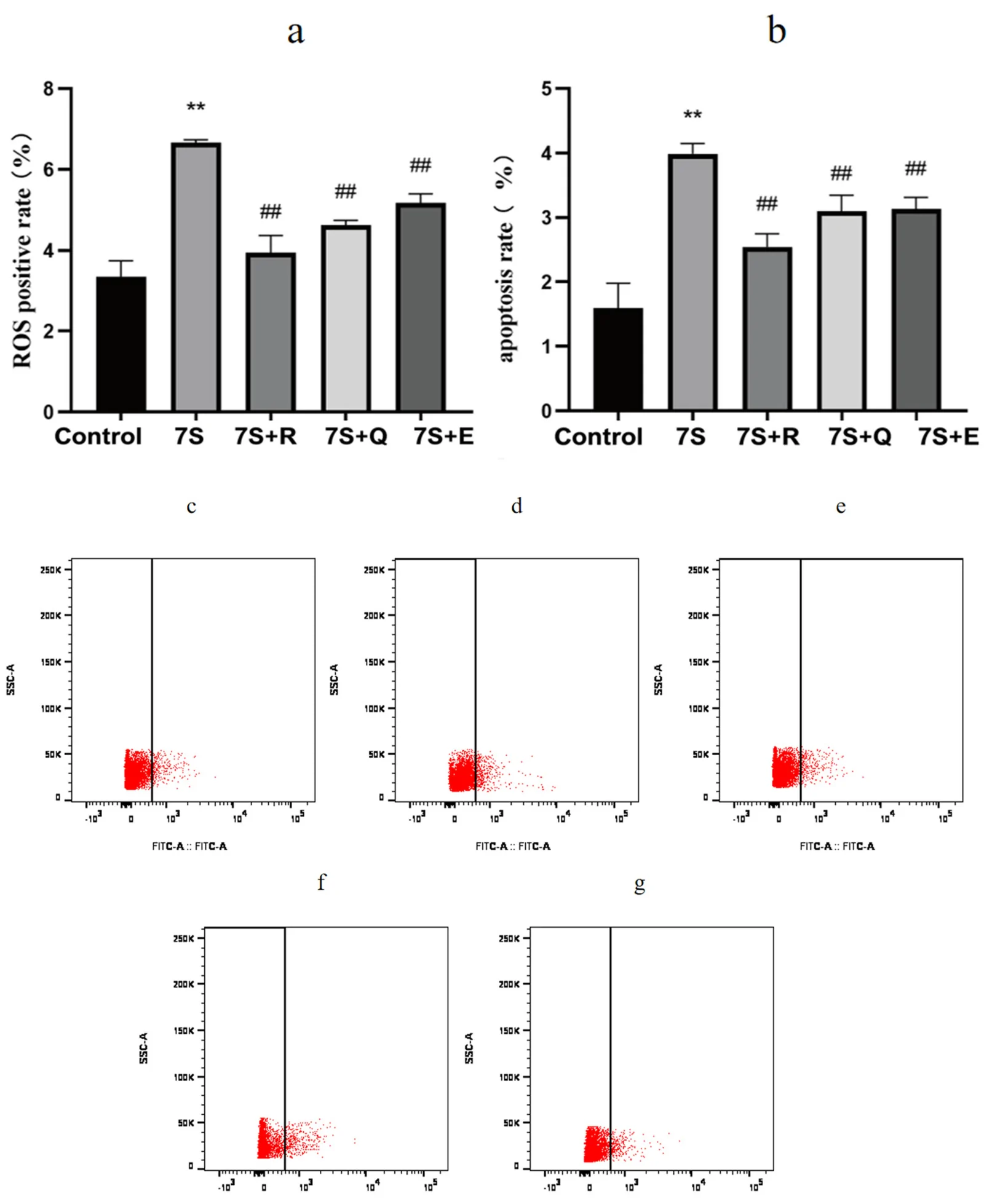

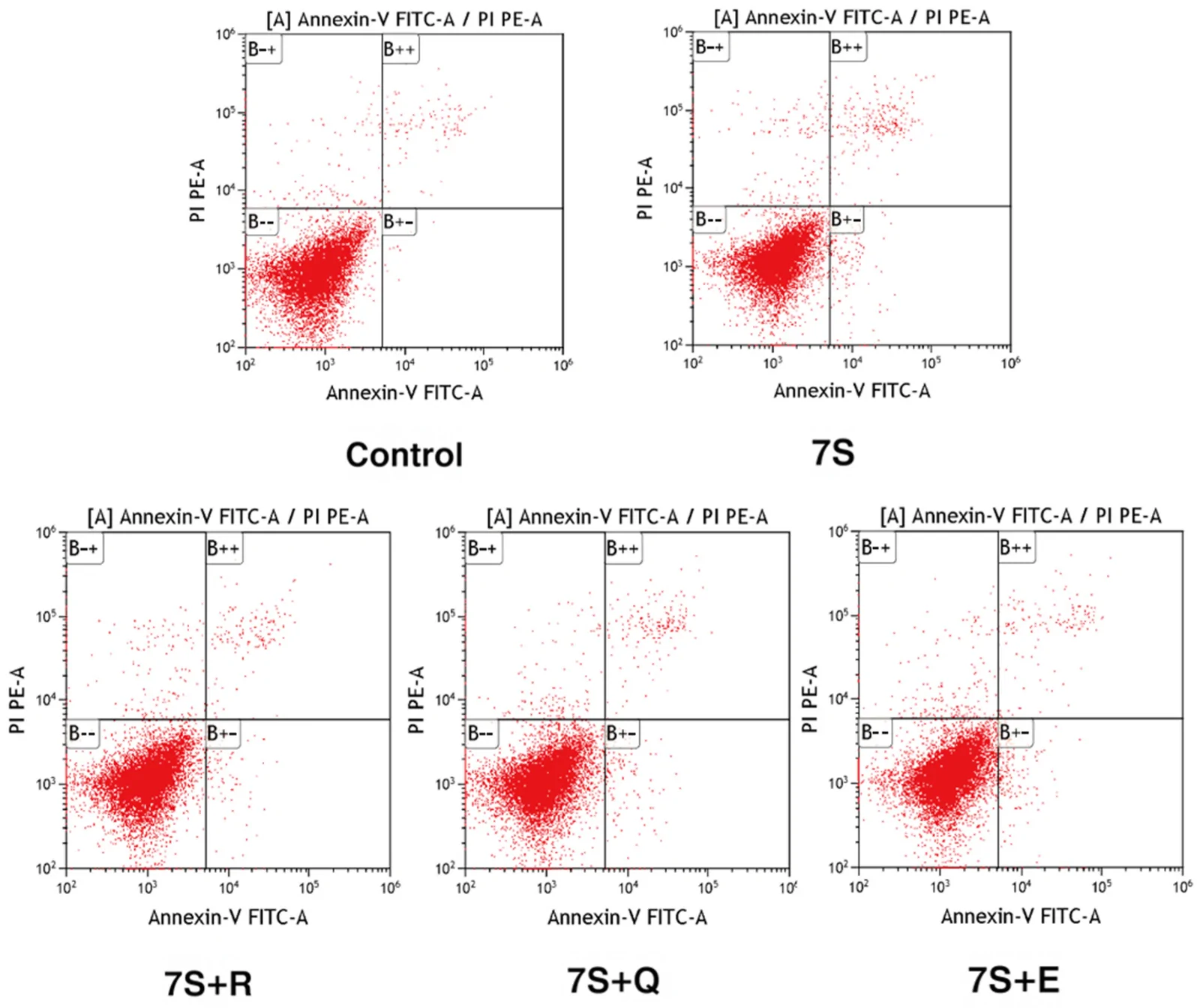
Effects of resveratrol, quercetin, and esculin on intracellular ROS generation and apoptosis in IPEC-J2 cells induced by soybean 7S globulin. Note: Representative flow cytometric scatter plots of intracellular ROS (**c**–**g**) and cell apoptosis were obtained by DCFH-DA staining and Annexin V-FITC/PI double-staining, respectively. (**a**) Bar chart of the ROS-positive cell rate; (**b**) bar chart of the total cell apoptosis rate. (**c**) Control group; (**d**) 7S group (5 mg/mL soybean 7S globulin); (**e**) 7S + Q group (7S globulin + 2.5 mg/L quercetin); (**f**) 7S + E group (7S globulin + 20 mg/L esculin); (**g**) 7S + R group (7S globulin + 0.625 mg/L resveratrol). In the scatter plots, each red dot represents one cell, and the vertical and horizontal lines indicate the thresholds distinguishing fluorescence-negative from fluorescence-positive populations; the quadrant symbols indicate Annexin V−/PI− (−−, live cells), Annexin V+/PI− (+−, early apoptotic cells), Annexin V−/PI+ (−+, necrotic or damaged cells), and Annexin V+/PI+ (++, late apoptotic cells). DCFH-DA probe was applied to detect intracellular ROS levels; Annexin V-FITC/PI double staining was used for apoptosis detection. Values are expressed as mean ± SD. ** *p* < 0.01 versus the control group; ## *p* < 0.01 versus the 7S group.

Intracellular reactive oxygen species (ROS) accumulation and cell apoptosis were determined by flow cytometry with DCFH-DA and Annexin V-FITC/PI double staining, respectively, and the results are presented in Figure 5. As shown in the scatter plots of Figure 5c–g, compared with the control group, treatment with 5 mg/mL soybean 7S globulin significantly elevated intracellular ROS fluorescence intensity and the proportion of ROS-positive IPEC-J2 cells (*p* < 0.01), indicating that soybean 7S globulin triggered excessive ROS production and induced intracellular oxidative stress. After co-incubation with 2.5 mg/L quercetin, 20 mg/L esculin and 0.625 mg/L resveratrol, the proportion of ROS-positive cells was markedly reduced relative to the 7S model group (*p* < 0.05 or *p* < 0.01).

Consistent with the changes in ROS levels, the apoptosis rate of IPEC-J2 cells was significantly increased under soybean 7S globulin stimulation (*p* < 0.01). Supplementation with quercetin, esculin, and resveratrol effectively mitigated 7S-provoked cell apoptosis, and the total apoptotic rate was significantly decreased in each compound intervention group compared with the 7S group (*p* < 0.05 or *p* < 0.01). Collectively, these observations suggested that resveratrol, quercetin and esculin could alleviate soybean 7S globulin-caused oxidative damage and apoptosis in IPEC-J2 cells via restraining over-accumulation of intracellular ROS.

### 3.6. Effects of Resveratrol, Quercetin, and Esculin on the mRNA Expression of Apoptosis-Related Genes in IPEC-J2 Cells Induced by Soybean 7S Globulin

**Figure 6:**
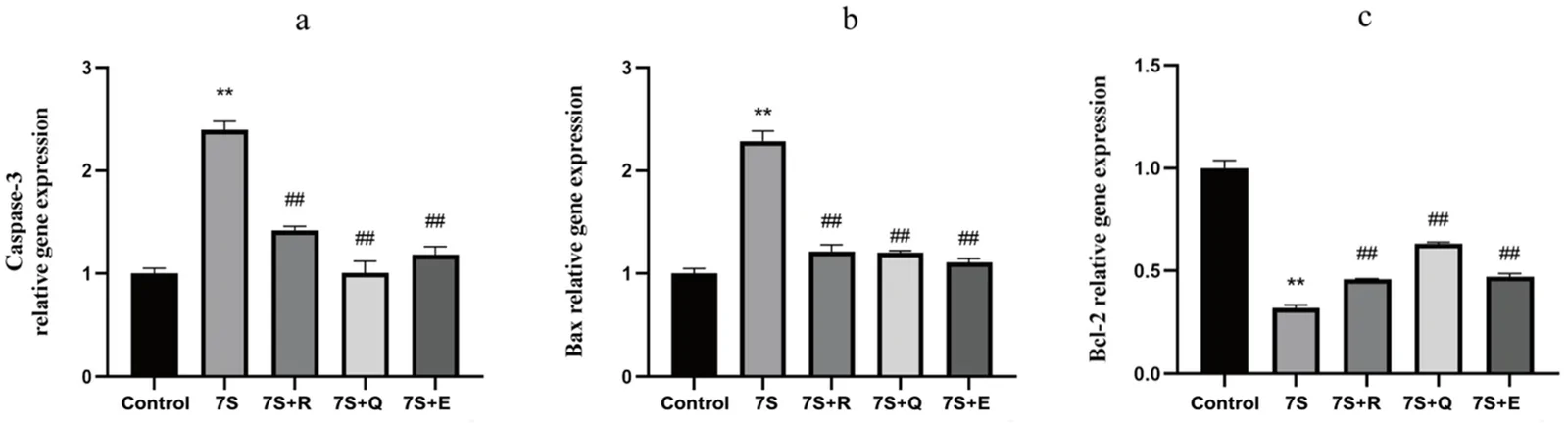
Effects of resveratrol, quercetin, and esculin on the mRNA expression of apoptosis-related genes in IPEC-J2 cells induced by soybean 7S globulin. Note: (**a**) Caspase-3 relative gene expression; (**b**) Bax relative gene expression; (**c**) Bcl-2 relative gene expression. ** *p* < 0.01 vs. the control group; ## *p* < 0.01 vs. the soybean 7S globulin model group.

The effects on the mRNA expression of apoptosis-related genes are shown in Figure 6. Compared with the control group, soybean 7S globulin significantly upregulated the mRNA expression of the pro-apoptotic genes Bax and Caspase-3 (both *p* < 0.01) and downregulated the mRNA expression of the anti-apoptotic gene Bcl-2 (*p* < 0.01). Compared with the soybean 7S globulin group, treatment with 0.625 mg/L resveratrol, 2.5 mg/L quercetin, or 20 mg/L esculin significantly downregulated Bax and Caspase-3 mRNA levels (*p* < 0.05 or *p* < 0.01) and upregulated Bcl-2 mRNA levels (*p* < 0.05 or *p* < 0.01).

### 3.7. Effects of Resveratrol, Quercetin, and Esculin on the Expression of NF-κB/MAPK Signaling Pathway-Related Proteins and mRNAs in IPEC-J2 Cells Induced by Soybean 7S Globulin

**Figure 7:**
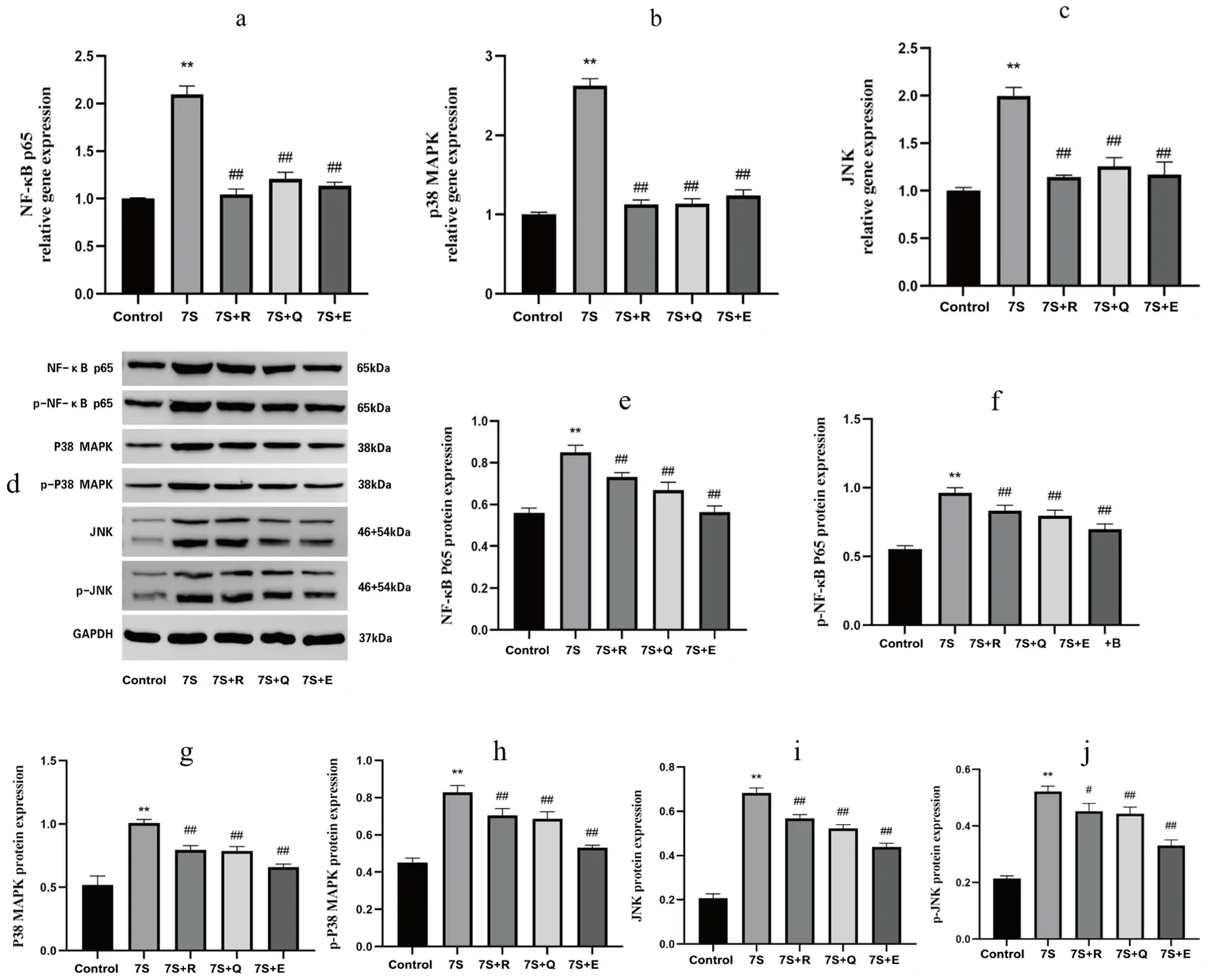
Effects of resveratrol, quercetin, and esculin on the expression of NF-κB/MAPK signaling pathway-related proteins and mRNAs in IPEC-J2 cells induced by soybean 7S globulin (the original Western blotting pictures can be found in File S1). Note: (**a**–**c**) Relative mRNA expression of NF-κB p65 (**a**), p38 MAPK (**b**), and JNK (**c**); (**d**) representative Western blotting bands; (**e**–**j**) protein expression of NF-κB p65 (**e**), p-NF-κB p65 (**f**), p38 MAPK (**g**), p-p38 MAPK (**h**), JNK (**i**), and p-JNK (**j**). ** *p* < 0.01 vs. the control group; # *p* < 0.05 and ## *p* < 0.01 vs. the soybean 7S globulin model group.

The effects on the mRNA and protein expression of NF-κB/MAPK pathway components are shown in Figure 7. At the mRNA level, soybean 7S globulin significantly upregulated the expression of NF-κB p65, p38 MAPK, and JNK compared with the control group (all *p* < 0.01), whereas all three compounds significantly downregulated the mRNA levels of these genes compared with the soybean 7S globulin group (*p* < 0.05 or *p* < 0.01).

At the protein level, there were no significant differences in the total protein levels of NF-κB p65, p38 MAPK, and JNK among the groups. Compared with the control group, soybean 7S globulin significantly increased the phosphorylation levels of NF-κB p65, p38 MAPK, and JNK (all *p* < 0.01). Compared with the soybean 7S globulin group, treatment with 0.625 mg/L resveratrol, 2.5 mg/L quercetin, or 20 mg/L esculin significantly suppressed the phosphorylation of these three proteins (*p* < 0.05 or *p* < 0.01).

## 4. Discussion

Soybean 7S globulin is a major allergenic protein in soybean-based feed and is a leading cause of allergic enteritis and diarrhea in weaned piglets. In the present study, we established an in vitro injury model in IPEC-J2 cells [27] using 5 mg/mL soybean 7S globulin and evaluated the protective effects of three bioactive phytochemicals—resveratrol, quercetin, and esculin. The principal findings of this study were that soybean 7S globulin induced oxidative stress, inflammatory responses, and apoptosis in IPEC-J2 cells, accompanied by activation of the NF-κB/MAPK signaling pathway, and that all three compounds alleviated these alterations to varying degrees. To our knowledge, this is the first study to systematically compare the protective effects of resveratrol, quercetin, and esculin against soybean 7S globulin-induced intestinal epithelial injury and to link these effects to the modulation of the NF-κB/MAPK pathway, which constitutes the main novelty and contribution of this work.

The intestine is an important immune organ, and oxidative stress is a key factor in the pathogenesis of gastrointestinal mucosal diseases [28]. Elevated ROS levels not only cause severe oxidative stress but also lead to lipid peroxidation, DNA damage, inflammation, and even cellular dysfunction, apoptosis, and death [29]. Intestinal ROS levels are closely associated with intestinal inflammation: ROS interact with target cell membrane components, causing lipid peroxidation, membrane disintegration, and endothelial cell damage, and can increase the expression of TNF, IL-1, and IL-6, promote the accumulation of pro-inflammatory mediators, and ultimately cause inflammatory damage to intestinal tissue [30]. Consistent with this mechanism, our results showed that soybean 7S globulin induced intracellular oxidative stress, as evidenced by an increased MDA concentration, reduced SOD activity, and a decreased GSH concentration, accompanied by elevated intracellular iNOS and NO concentrations. The three compounds tested reversed these changes, indicating that their protective effects are at least partly attributable to the attenuation of oxidative stress.

Oxidative stress is also intimately linked to apoptosis. Apoptosis is a form of programmed cell death regulated by anti-apoptotic factors (e.g., Bcl-2) and pro-apoptotic factors (e.g., p53, Bax, and Caspase-3) [31]. During oxidative stress, large amounts of ROS accumulate, causing lipid peroxidation and MDA production, oxidative damage to cellular proteins and DNA, protein denaturation, and disruption of biomembrane structure and function. Through the NF-κB/MAPK signaling pathway [32], these events upregulate the pro-apoptotic genes Caspase-3 and Bax, downregulate the anti-apoptotic gene Bcl-2, and increase the Bax/Bcl-2 protein ratio, ultimately triggering apoptosis [33]. In agreement with this mechanism, soybean 7S globulin upregulated the expression of the pro-apoptotic genes Caspase-3 and Bax, suppressed Bcl-2 expression, and increased the apoptosis rate in the present study, whereas resveratrol, quercetin, and esculin alleviated all of these changes. Our observations are consistent with previous reports: resveratrol has been shown to regulate intracellular ROS production by modulating the activation of the MAPK signaling pathway, thereby controlling the apoptotic process [34]; quercetin protects pig intestinal integrity after transport stress by regulating oxidative status and inflammation [35]; and esculin exerts anti-apoptotic effects by reducing ROS levels, preserving mitochondrial membrane potential, enhancing SOD activity, increasing GSH levels, and regulating the expression of p53, Bax, and Bcl-2 [36]. These three compounds may therefore inhibit apoptosis and inflammatory responses and restore cell viability by mitigating oxidative stress.

Beyond directly inducing inflammation and affecting apoptotic genes, oxidative stress also activates the transcription factor NF-κB. The NF-κB transcription factor complex is a cellular sensor of oxidative stress that regulates gene expression and is involved in stress, injury, and inflammatory pathways [37]. NF-κB activation is associated with increased oxidative damage, decreased GSH content, and elevated ROS levels [38]. The MAPK signaling pathway is another important intracellular signaling system that regulates cell growth, development, and apoptosis; JNK and p38 are activated by inflammatory cytokines and various cellular stress cascades [39]. Our results showed that soybean 7S globulin increased the expression of the NF-κB/MAPK pathway proteins NF-κB p65, p38 MAPK, and JNK and significantly increased their phosphorylation levels, consistent with the findings of Peng et al. [14]. These results indicate that soybean 7S globulin-induced oxidative stress promotes inflammatory responses and apoptosis in intestinal epithelial cells by activating the NF-κB/MAPK signaling pathway, thereby damaging the intestine. Resveratrol, quercetin, and esculin significantly downregulated the mRNA expression of NF-κB/MAPK pathway components and suppressed the phosphorylation of the corresponding proteins in IPEC-J2 cells, in line with previous reports. For example, Yang et al. [40] found that resveratrol alleviated hydrogen peroxide-induced oxidative stress, inflammatory cytokine secretion, and apoptosis in IPEC-J2 cells through the ERK1/2 and AKT signaling pathways; Wang et al. [41] reported that quercetin inhibited p38 MAPK and NF-κB expression in the kidney, reduced serum IL-8 levels, and alleviated renal inflammation in diabetic rats by modulating the NF-κB/MAPK pathway; and Li et al. [42] showed that esculin reduced iNOS, TNF-α, and IL-6 expression by inhibiting NF-κB activation, thereby attenuating ethanol-induced gastric inflammatory injury in mice.

Several limitations of this study should be acknowledged. First, the commercial soybean 7S globulin preparation used in this study had a purity of 83 ± 3% and may have contained trace impurities, including endotoxins, which could potentially contribute to NF-κB activation; therefore, interference from impurities in the cellular inflammatory response cannot be completely excluded, and further studies using higher-purity soybean 7S globulin are warranted to validate our findings. Second, this study relied solely on the IPEC-J2 monolayer cell model, and the in vitro results require confirmation in animal experiments. Third, the effective concentrations determined in this two-dimensional cell model (0.625 mg/L resveratrol, 2.5 mg/L quercetin, and 20 mg/L esculin) cannot be directly extrapolated to in vivo dosages in piglets. Although oral administration is the most feasible route for feed additives, the bioavailability and gastrointestinal metabolism of these compounds remain unclear. Finally, a single reference gene (β-actin) was used for qRT-PCR normalization, which, although validated for stability in our system, represents a methodological limitation. Future studies will focus on dose translation, pharmacokinetics, and pre-clinical validation in weaned piglets to confirm the translational value of these findings.

## 5. Conclusions

Soybean 7S globulin (5 mg/mL) induces oxidative stress in IPEC-J2 cells, promotes inflammatory cytokine secretion and lipid peroxidation, and dysregulates apoptosis-related gene expression, thereby causing inflammatory injury and apoptosis; these effects may be mediated by the NF-κB/MAPK signaling pathway. Resveratrol (0.625 mg/L), quercetin (2.5 mg/L), and esculin (20 mg/L) alleviate soybean 7S globulin-induced inflammatory injury by modulating the NF-κB/MAPK signaling pathway, thereby protecting intestinal epithelial health. These findings provide a theoretical basis for the development of these bioactive phytochemicals as natural feed additives for the prevention of soybean 7S globulin-induced allergic enteritis in weaned piglets.

## Figures and Tables

**Table 1:** Primer sequences used for quantitative real-time PCR. F, forward primer; R, reverse primer.

Name	Primer Sequence (5′ → 3′)
Caspase-3	(F) GTGGGACTGAAGATGACA
	(R) ACCCGAGTAAGAATGTG
Bcl-2	(F) ACCTGAATGACCACCTAGAGC
	(R) TCCGACTGAAGAGCGAAC
Bax	(F) CCGAAATGTTTGCTGACG
	(R) AGCCGATCTCGAAGGAAGT
NF-κB p65	(F) CTCGCTCGCACTCTTGCTCTC
	(R) AATAGGCGGACGAGGCAGAAG
P38 MAPK	(F) AAGACTCGTTGGAACCCCAG
	(R) TCCAGCAAGTCAACAGCCAA
JNK	(F) CAAGCACCTTCACTCTGCTG
	(R) TCAGGTGCTCTGTAGTAGCG
β-actin	(F) CTGGACTTCGAGCAGGAGATGG
	(R) TTCGTGGATGCCGCAGGATTC

## Data Availability

The original contributions presented in this study are included in the article/Supplementary Material. Further inquiries can be directed to the corresponding author.

## Supplementary Materials

The following supporting information can be downloaded at: https://www.mdpi.com/article/10.3390/vetsci13101048/s1. File S1: Original uncropped images of the Western blotting membranes shown in Figure 7.
